# Furfuryl­ammonium chloridozincophosphate

**DOI:** 10.1107/S1600536810029995

**Published:** 2010-07-31

**Authors:** Kamel Kaabi, Meher El Glaoui, Erwann Jeanneau, Frederic Lefebvre, Cherif Ben Nasr

**Affiliations:** a Laboratoire de Chimie des Matériaux, Faculté des Sciences de Bizerte, 7021 Zarzouna, Tunisia; b Universtié Lyon1, Centre de Diffractométrie Henri Longchambon, 43 boulevard du 11 Novembre 1918, 69622 Villeurbanne Cedex, France; c Laboratoire de Chimie Organometallique de Surface (LCOMS), Ecole Superieure de Chimie Physique Electronique, 69622 Villeurbanne Cedex, France

## Abstract

In the title compound, [ZnCl(HPO_4_)](C_5_H_8_NO), polymeric inorganic layers constructed from ZnO_3_Cl and PO_4_ tetra­hedra are linked by O atoms: O—H⋯O hydrogen bonds occur within the layers.  The organic cations occupy the interlayer regions and interact with the layers by way of N—H⋯O, N—H⋯Cl,  and C—H⋯Cl hydrogen bonds.

## Related literature

For related zincophosphate materials, see: Gier & Stucky (1991 ▶); Harrison & Phillips (1997 ▶). For a discussion of Zn—O and P—O distances, see: Rayes *et al.* (2001 ▶); Kefi *et al.* (2007 ▶). For the Chebychev weighting scheme, see: Prince (1982 ▶); Watkin (1994 ▶). 
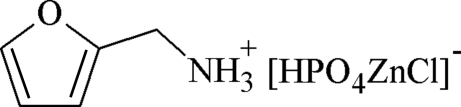


## Experimental

### 

#### Crystal data

[ZnCl(HPO_4_)](C_5_H_8_NO)
*M* *_r_* = 294.94
Monoclinic, 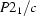

*a* = 12.7588 (4) Å
*b* = 9.6339 (2) Å
*c* = 8.6281 (2) Å
β = 106.233 (3)°
*V* = 1018.26 (5) Å^3^
*Z* = 4
Mo *K*α radiation
μ = 2.83 mm^−1^
*T* = 293 K
0.36 × 0.15 × 0.08 mm

#### Data collection

Oxford Diffraction Xcalibur Eos Nova diffractometer
Absorption correction: analytical (*CrysAlis PRO*; Oxford Diffraction, 2009 ▶) *T*_min_ = 0.488, *T*_max_ = 0.806
7978 measured reflections
2409 independent reflections
1997 reflections with *I* > 2.0σ(*I*)
*R*_int_ = 0.024
2 standard reflections every 400 reflections  intensity decay: 4%

#### Refinement

*R*[*F* ^2^ > 2σ(*F* ^2^)] = 0.025
*wR*(*F* ^2^) = 0.042
*S* = 1.01
2409 reflections
127 parameters
H-atom parameters constrained
Δρ_max_ = 0.54 e Å^−3^
Δρ_min_ = −0.55 e Å^−3^

### 

Data collection: *CrysAlis PRO* (Oxford Diffraction, 2009 ▶); cell refinement: *CrysAlis PRO*; data reduction: *CrysAlis PRO*; program(s) used to solve structure: *SIR97* (Altomare *et al.*, 1999 ▶); program(s) used to refine structure: *CRYSTALS* (Betteridge *et al.*, 2003 ▶); molecular graphics: *CAMERON* (Watkin *et al.*, 1996 ▶); software used to prepare material for publication: *CRYSTALS* .

## Supplementary Material

Crystal structure: contains datablocks global, I. DOI: 10.1107/S1600536810029995/bx2290sup1.cif

Structure factors: contains datablocks I. DOI: 10.1107/S1600536810029995/bx2290Isup2.hkl

Additional supplementary materials:  crystallographic information; 3D view; checkCIF report

## Figures and Tables

**Table 1:** Selected bond lengths (Å)

Zn1—O3^i^	1.9637 (16)
Zn1—O2^ii^	1.9368 (16)
Zn1—Cl1	2.2161 (8)
Zn1—O1	1.9416 (16)
P1—O1	1.5150 (18)
P1—O2	1.5218 (17)
P1—O3	1.5187 (16)
P1—O4	1.5699 (17)

Symmetry codes: (i) 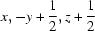; (ii) 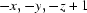.

**Table 2:** Hydrogen-bond geometry (Å, °)

*D*—H⋯*A*	*D*—H	H⋯*A*	*D*⋯*A*	*D*—H⋯*A*
O4—H1⋯O2^i^	0.80	1.91	2.709 (2)	177
N1—H4⋯O1	0.89	2.28	3.051 (6)	145
N1—H3⋯O3^iv^	0.91	1.99	2.896 (6)	172
N1—H2⋯Cl1^iii^	0.92	2.35	3.234 (3)	161
C5—H9⋯Cl1	0.96	2.87	3.640 (3)	138

Symmetry codes: (i) 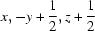; (iii) 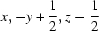; (iv) 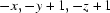.

**Fig. 1.**
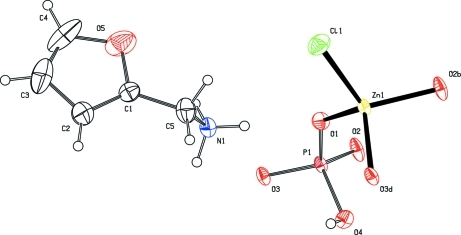
View of (I), showing 50% probability displacement ellipsoids and arbitrary spheres for the H atoms. For the disordered perchlorate anions, only major parts are shown. Dashed lines denote hydrogen bonds. [Symmetry codes: (b) -x, -y, 1-z; (d) x, 1/2-y, 1/2+z]

**Fig. 2.**
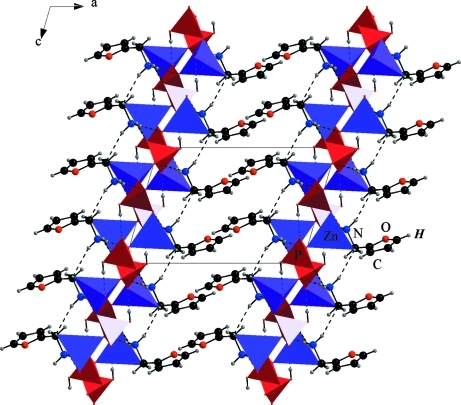
Polyhedral representation of the framework Zn(HPO4)ClC5H5ONH3, viewed the a direction.

**Fig. 3.**
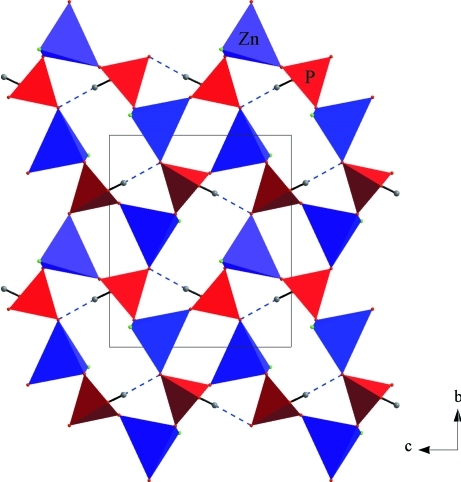
Projection of Zn(HPO4)ClC5H5ONH3 structure in the plane (a, c). The hydrogen bonds are denoted by dotted lines.

## supplementary crystallographic information

### Comment

Recently, zincophosphates with monomeric phases, chains, layers and three-dimensional open framework have been prepared in the presence of different amines, alkali metal cations or metal complexes as structure directing agent (Gier and Stucky, 1991; Harrison and Phillips, 1997). We report here the crystal structure of one such compound, Zn(HPO_4_)ClC_5_H_5_ONH_3_ (I), (Fig. 1). The atomic arrangement of the title compound consists of corrugated anionic layers of formula [Zn(HPO_4_)Cl]_n_^n-^ parallel to (b, c) plane. Charge neutrality is achieved by, the presence of protonated furfurylamine templete cation trapped in the inter-layer spacing (Fig. 2). Both zinc and phosphorus atoms are tetrahedrally coordinated. The zinc atom is connected by three phosphate groups and has one terminal Zn—Cl vertex. On the other hand, each phosphorus atom is bonded to three Zn atoms through three oxygen atoms with the forth coordination site being a terminal P—OH group. The topology of the zincophosphate connectivity pattern is shown in Fig. 3.

The ZnO_3_Cl and PO_4_ groups in Zn(HPO_4_)ClC_5_H_5_ONH_3_ fuse together *via* Zn—O—P bridges lead to a two-dimensional network. The resulting infinite anionic layers parallel to (b, c) plane are situated at *x* = 0. These layers are arranged in such away as to create two kinds of pores. The first one, built up from four-membered [Zn_2_P_2_] rings (presents an approximate dimensions 4.426 × 3.911 Å) and the second one formed by eight-membered [Zn_4_P_4_] rings (exhibits as approximate dimensions 9.571 × 3.376 Å)) This inorganic framework, with a 4.8^2^ topology, is closely similar to that of Zn(HPO_4_)ClC_5_H_12_N [24]. However, these second pores are not completely accessible due to the presence of P—OH groups extending into them, thereby blocking the entry to pores (Fig. 2). In the [Zn(HPO_4_)Cl]_n_^n-^ layers, the bond-length values (Zn—O (mean= 1.947 (2) Å, Zn—Cl = 2.216 (1) Å and P—O(mean) = 1.531 (2) Å) are close to those observed in other zincophosphate containing similar polyhedron Zn(HPO_4_)ClC_5_H_12_N (Rayes *et al.*, 2001) and Zn(HPO_4_)ClC_4_H_10_NO (Kefi *et al.*, 2007). Among the four distinct oxygen of the PO_3_OH) unit, three are bonded with Zn atoms, while the other has a significantly longer bond length (P—O = 1.570 (2) Å) suggesting that oxygen O(1) is an hydroxyl group atom. Hydrogen bonds plays an important role in stabilizing the Zn(HPO_4_)ClC_5_H_5_ONH_3_ structure. Furfurylaminium cations interact with zincophosphate layers through N—H···O and N—H···Cl hydrogen bonds. Inside layers, the P—O—H groups are interconnected *via* O—H···O hydrogen bonds (Fig. 3).

### Experimental

The title compound Zn(HPO_4_)ClC_5_H_5_ONH_3_ was prepared at room temperature by adding 5.8 g (50 mmol) of orthophosphoric acid (85 weight % from Fluka) to a solution of 4.8 g of furfurylamine (50 mmol)(Acros) in 60 ml of water. To this mixture, we added, drop by drop, an aqueous solution of 6.8 g (50 mmol) of zinc chloride (Prolabo) under continuous stirring. A white precipitate was formed which completely dissolved by adding phosphoric acid. The obtained solution was slowly evaporated at room temperature until the formation of needle colorless crystals of the title compound (yield 53%).

### Refinement

The H atoms were all located in a difference map, but those attached to carbon atoms were repositioned geometrically. The H atoms were initially refined with soft restraints on the bond lengths and angles to regularize their geometry (C—H in the range 0.93–0.98, N—H in the range 0.86–0.89 N—H to 0.86 O—H = 0.82 Å) and *U*_iso_(H) (in the range 1.2–1.5 times *U*_eq_ of the parent atom), after which the positions were refined with riding constraints.

### Crystal data

**Table:**

[ZnCl(HPO_4_)](C_5_H_8_NO)	*F*(000) = 592
*M**_r_* = 294.94	*D*_x_ = 1.924 Mg m^−^^3^
Monoclinic, *P*2_1_/*c*	Mo *K*α radiation, λ = 0.71073 Å
Hall symbol: -P 2ybc	Cell parameters from 6031 reflections
*a* = 12.7588 (4) Å	θ = 2.5–29.1°
*b* = 9.6339 (2) Å	µ = 2.83 mm^−^^1^
*c* = 8.6281 (2) Å	*T* = 293 K
β = 106.233 (3)°	Needle, colorless
*V* = 1018.26 (5) Å^3^	0.36 × 0.15 × 0.08 mm
*Z* = 4	

### Data collection

**Table:**

Oxford Diffraction Xcalibur Eos Nova diffractometer	1997 reflections with *I* > 2.0σ(*I*)
Radiation source: Mova (Mo) X-ray Source	*R*_int_ = 0.024
mirror	θ_max_ = 29.2°, θ_min_ = 3.2°
ω scans	*h* = −17→17
Absorption correction: analytical (*CrysAlis PRO*; Oxford Diffraction, 2009)	*k* = −12→11
*T*_min_ = 0.488, *T*_max_ = 0.806	*l* = −11→11
7978 measured reflections	2 standard reflections every 400 reflections
2409 independent reflections	intensity decay: 4%

### Refinement

**Table:**

Refinement on *F*^2^	Hydrogen site location: inferred from neighbouring sites
Least-squares matrix: full	H-atom parameters constrained
*R*[*F*^2^ > 2σ(*F*^2^)] = 0.025	Method, part 1, Chebychev polynomial, (Watkin, 1994, *P*rince, 1982) [*w*eight] = 1.0/[A_0_*T_0_(x) + A_1_*T_1_(x) ··· + A_n-1_]*T_n-1_(x)] where A_i_ are the Chebychev coefficients listed belo*w* and x = *F* /*F*max Method = Robust Weighting (*P*rince, 1982) W = [*w*eight] * [1-(delta*F*/6*sigma*F*)^2^]^2^ A_i_ are: 0.105E + 04 0.146E + 04 711. 140. -26.6
*wR*(*F*^2^) = 0.042	(Δ/σ)_max_ = 0.001
*S* = 1.01	Δρ_max_ = 0.54 e Å^−^^3^
2409 reflections	Δρ_min_ = −0.55 e Å^−^^3^
127 parameters	Extinction correction: Larson (1970), Equation 22
0 restraints	Extinction coefficient: 0.000
Primary atom site location: structure-invariant direct methods	

### Special details

**Table:**

Experimental. CrysAlisPro, Oxford Diffraction Ltd., Version 1.171.33.52 (release 06-11-2009 CrysAlis171 .NET) (compiled Nov 6 2009,16:24:50) Analytical numeric absorption correction using a multifaceted crystal model based on expressions derived by R.C. Clark & J.S. Reid. (Clark, R. C. & Reid, J. S. (1995). Acta Cryst. A51, 887-897)The crystal was placed in the cold stream of an Oxford Cryosystems open-flow nitrogen cryostat (Cosier & Glazer, 1986) with a nominal stability of 0.1 K.Cosier, J. & Glazer, A.*M*., 1986. J. Appl. Cryst. 105 107.

### Fractional atomic coordinates and isotropic or equivalent isotropic displacement parameters (Å^2^)

**Table:**

	*x*	*y*	*z*	*U*_iso_*/*U*_eq_	
Zn1	0.09697 (2)	0.06480 (3)	0.74519 (3)	0.0229	
Cl1	0.26982 (6)	0.09461 (9)	0.88474 (10)	0.0500	
O1	0.07464 (14)	0.17891 (18)	0.5532 (2)	0.0311	
P1	−0.02439 (5)	0.22912 (6)	0.42328 (7)	0.0209	
O2	−0.05740 (16)	0.12965 (16)	0.28087 (19)	0.0333	
O3	−0.00551 (14)	0.37260 (16)	0.36327 (19)	0.0258	
O4	−0.12558 (14)	0.23883 (19)	0.4926 (2)	0.0307	
O5	0.4270 (2)	0.5428 (4)	0.7914 (3)	0.0841	
C1	0.3492 (2)	0.5791 (3)	0.8597 (3)	0.0405	
C2	0.3614 (3)	0.7099 (4)	0.9043 (5)	0.0698	
C3	0.4544 (4)	0.7592 (6)	0.8687 (6)	0.0941	
C4	0.4910 (4)	0.6607 (7)	0.8001 (5)	0.0998	
C5	0.2697 (3)	0.4722 (3)	0.8692 (4)	0.0518	
N1	0.19146 (18)	0.4419 (3)	0.7080 (3)	0.0436	
H1	−0.1048	0.2746	0.5792	0.0473*	
H2	0.2282	0.4435	0.6304	0.0669*	
H3	0.1376	0.5071	0.6866	0.0666*	
H4	0.1624	0.3575	0.7093	0.0668*	
H5	0.3184	0.7576	0.9503	0.0857*	
H6	0.4834	0.8469	0.8901	0.1143*	
H7	0.5500	0.6638	0.7591	0.1243*	
H8	0.2309	0.4976	0.9458	0.0636*	
H9	0.3096	0.3879	0.9034	0.0635*	

### Atomic displacement parameters (Å^2^)

**Table:**

	*U*^11^	*U*^22^	*U*^33^	*U*^12^	*U*^13^	*U*^23^
Zn1	0.03217 (15)	0.01510 (12)	0.02197 (13)	−0.00057 (12)	0.00852 (11)	0.00072 (11)
Cl1	0.0318 (4)	0.0606 (5)	0.0526 (4)	−0.0051 (4)	0.0034 (3)	0.0016 (4)
O1	0.0349 (10)	0.0312 (10)	0.0282 (9)	0.0045 (8)	0.0107 (8)	0.0126 (8)
P1	0.0343 (3)	0.0148 (3)	0.0160 (3)	−0.0006 (2)	0.0111 (2)	0.0004 (2)
O2	0.0672 (14)	0.0159 (8)	0.0209 (8)	−0.0060 (8)	0.0189 (9)	−0.0030 (7)
O3	0.0410 (10)	0.0161 (8)	0.0234 (8)	−0.0004 (7)	0.0143 (7)	0.0020 (6)
O4	0.0341 (10)	0.0373 (10)	0.0239 (8)	−0.0036 (8)	0.0135 (8)	−0.0039 (8)
O5	0.0689 (19)	0.109 (2)	0.086 (2)	−0.0059 (18)	0.0394 (16)	−0.0313 (19)
N1	0.0384 (13)	0.0287 (12)	0.0622 (16)	−0.0040 (11)	0.0115 (12)	−0.0030 (12)
C1	0.0363 (15)	0.0487 (18)	0.0349 (14)	−0.0017 (14)	0.0073 (12)	−0.0030 (14)
C2	0.063 (2)	0.051 (2)	0.101 (3)	−0.0071 (19)	0.032 (2)	−0.020 (2)
C3	0.085 (4)	0.090 (4)	0.101 (4)	−0.047 (3)	0.015 (3)	−0.004 (3)
C4	0.051 (3)	0.183 (6)	0.070 (3)	−0.042 (3)	0.024 (2)	−0.007 (4)
C5	0.062 (2)	0.0439 (19)	0.0475 (18)	−0.0063 (16)	0.0113 (16)	0.0055 (15)

### Geometric parameters (Å, °)

**Table:**

Zn1—O3^i^	1.9637 (16)	C1—C2	1.315 (5)
Zn1—O2^ii^	1.9368 (16)	C5—N1	1.497 (4)
Zn1—Cl1	2.2161 (8)	C5—H9	0.960
Zn1—O1	1.9416 (16)	C5—H8	0.961
O1—P1	1.5150 (18)	N1—H4	0.895
P1—O2	1.5218 (17)	N1—H3	0.911
P1—O3	1.5187 (16)	N1—H2	0.918
P1—O4	1.5699 (17)	C2—C3	1.390 (5)
O4—H1	0.798	C2—H5	0.889
O5—C1	1.336 (4)	C3—C4	1.274 (7)
O5—C4	1.389 (6)	C3—H6	0.920
C1—C5	1.463 (4)	C4—H7	0.917
			
O3^i^—Zn1—O2^ii^	99.65 (7)	C1—C5—H9	107.2
O3^i^—Zn1—Cl1	112.59 (6)	N1—C5—H9	106.2
O2^ii^—Zn1—Cl1	112.08 (6)	C1—C5—H8	110.9
O3^i^—Zn1—O1	107.97 (7)	N1—C5—H8	110.6
O2^ii^—Zn1—O1	118.51 (7)	H9—C5—H8	109.6
Cl1—Zn1—O1	106.05 (6)	C5—N1—H4	109.6
Zn1—O1—P1	134.84 (11)	C5—N1—H3	108.8
O1—P1—O2	112.44 (11)	H4—N1—H3	109.7
O1—P1—O3	111.34 (10)	C5—N1—H2	109.3
O2—P1—O3	109.41 (9)	H4—N1—H2	108.8
O1—P1—O4	109.99 (10)	H3—N1—H2	110.5
O2—P1—O4	105.88 (10)	C1—C2—C3	107.6 (4)
O3—P1—O4	107.53 (10)	C1—C2—H5	126.1
Zn1^ii^—O2—P1	135.00 (10)	C3—C2—H5	126.4
Zn1^iii^—O3—P1	130.43 (10)	C2—C3—C4	107.2 (4)
P1—O4—H1	106.8	C2—C3—H6	126.1
C1—O5—C4	105.1 (3)	C4—C3—H6	126.7
O5—C1—C5	117.0 (3)	O5—C4—C3	110.4 (4)
O5—C1—C2	109.8 (3)	O5—C4—H7	122.6
C5—C1—C2	133.3 (3)	C3—C4—H7	127.1
C1—C5—N1	112.2 (2)		

Symmetry codes: (i) *x*, −*y*+1/2, *z*+1/2; (ii) −*x*, −*y*, −*z*+1; (iii) *x*, −*y*+1/2, *z*−1/2.

### Hydrogen-bond geometry (Å, °)

**Table:**

*D*—H···*A*	*D*—H	H···*A*	*D*···*A*	*D*—H···*A*
O4—H1···O2^i^	0.80	1.91	2.709 (2)	177
N1—H4···O1	0.89	2.28	3.051 (6)	145
N1—H3···O3^iv^	0.91	1.99	2.896 (6)	172
N1—H2···Cl1^iii^	0.92	2.35	3.234 (3)	161
C5—H9···Cl1	0.96	2.87	3.640 (3)	138

Symmetry codes: (i) *x*, −*y*+1/2, *z*+1/2; (iv) −*x*, −*y*+1, −*z*+1; (iii) *x*, −*y*+1/2, *z*−1/2.
